# Case Report: Dialysis following sintilimab-induced stage 3 acute kidney injury: mechanism investigation and management strategies

**DOI:** 10.3389/fonc.2025.1632740

**Published:** 2025-10-20

**Authors:** Bin Shan, Mengjiao Li, Ruixia Yang, Guanqi Wang, Juan Hou, Tianjiao Wen

**Affiliations:** Department of Pharmacy, The Fourth Hospital of Hebei Medical University, Shijiazhuang, Hebei, China

**Keywords:** sintilimab, acute kidney injury, dialysis, immune checkpoint inhibitors, PD-1 inhibitor, adverse reactions, literature review

## Abstract

Immune checkpoint inhibitors (ICIs) are widely used in the treatment of various tumor types. ICIs kill tumor cells by activating the body’s immune function. As this action is nonspecific, it inevitably triggers immune-related adverse events (irAEs), which can affect virtually all organs. Although the renal toxicity associated with ICIs such as pembrolizumab, nivolumab, and ipilimumab has been studied, research on domestically developed Chinese ICIs, including camrelizumab, sintilimab, tislelizumab, and toripalimab, remains limited. This paper presents the case of a lung cancer patient who developed stage 3 acute kidney injury (AKI) requiring dialysis following treatment with sintilimab. A literature review suggests that this is likely the second documented case of AKI necessitating dialysis after a single dose of sintilimab. Early recognition of irAEs, identification of risk factors, regular monitoring, steroid administration, and supportive care are crucial for improving patient outcomes. It should be emphasized that the tumor benefits of ICI therapy outweigh the risks of ICI-induced renal injury. In such cases, ICI treatment should not be discontinued or delayed, except in rare circumstances such as acute renal failure. When AKI occurs, healthcare professionals must be familiar with renal-related irAEs in order to facilitate the effective diagnosis and management of this increasingly common renal complication.

## Introduction

ICIs primarily comprise cytotoxic T lymphocyte-associated antigen 4 (CTLA-4) inhibitors and programmed cell death protein 1 (PD-1)/programmed death-ligand 1/2 (PD-L1/PD-L2) inhibitors. PD-1/PD-L1 inhibitors are more widely used in clinical practice.PD-1 and PD-L1/PD-L2 inhibitors block the negative regulatory signals that suppress the immune system in T cells. While increasing the antitumor effect of T cells, they may also abnormally enhance their own normal immune response, leading to an imbalance of immune tolerance as well as immune-related adverse events (irAEs) when normal tissues are affected (1). Such adverse events in the skin, intestines, endocrine system, lungs, and musculoskeletal system are relatively common, while irAEs in the cardiovascular system, blood, kidneys, nerves, and eyes are less common. Acute kidney injury (AKI) is a rare adverse reaction of an immune checkpoint inhibitor (ICI) (2).

Developed by Innovent Biologics in collaboration with Eli Lilly and Company, sintilimab is a novel humanized IgG4 monoclonal antibody that was launched in China in December 2018. When administered in combination with chemotherapy, sintilimab is indicated as a first-line treatment for patients with locally advanced or metastatic squamous non-small cell lung cancer (NSCLC) that is unresectable. It is also indicated for the treatment of relapsed or refractory classical Hodgkin lymphoma, hepatocellular carcinoma, esophageal carcinoma, and other conditions. At present, one case of sintimilab administration resulting in AKI and requiring dialysis treatment has been reported (8). AKI is commonly stage 1–2 (3–7), and there are very few reports of stage 3 AKI. This present article reports the second case of stage 3 AKI requiring dialysis treatment after sintilimab administration and offers a literature review of this reaction. The patient’s main clinical manifestations were fatigue and oliguria, blood potassium and creatinine were elevated, the evaluation was stage 3 AKI, and the adverse reaction was grade 4. Sintilimab was discontinued to reduce potassium and improve renal blood circulation, dialysis, glucocorticoids, and other treatments. After the patient’s creatinine level decreased, their electrolytes were normal, dialysis was stopped, and the patient was discharged from the hospital without any discomfort. The creatinine of later follow-up patients did not return to normal. Through a literature analysis, this paper presents the second case of AKI dialysis treatment after a single dose of sintilimab. This article details the case characteristics, pathological types, and molecular mechanisms of AKI caused by sintilimab. It aims to recommend that the clinical application of immune checkpoint inhibitors should involve close monitoring of kidney function, improved early diagnosis and prognosis of AKI with the help of new biological markers, and enhanced drug safety of ICIs.

## Clinical case

A 71-year-old male patient underwent coronary stent implantation in 2020 following an acute myocardial infarction. Following the procedure, he was prescribed aspirin (100 mg po qd) and clopidogrel sulfate (75 mg po qd). In the same year, he was diagnosed with hypertension, and his blood pressure was controlled at about 115/80 mmHg. In February and September 2022, tracheoscopy revealed a new lesion in the opening of the left upper lobe and that the opening of the upper lobe was completely blocked; the pathology indicated squamous cell carcinoma. On September 6, 2022, Positron Emission Tomography/Computed Tomography(PET/CT) showed an irregular soft tissue mass in the left hilar with uneven high metabolism and a solid change in the anterior segment of the upper lobe accompanied by slightly high metabolism. The patient presented with central lung carcinoma in the left lung, accompanied by atelectasis of the anterior segment of the upper lobe. According to the Ninth Edition of the International Association for the Study of Lung Cancer, the TNM staging was T2aN0M0. On September 18, 2022, a new preoperative auxiliary treatment was given, with 400 mg of paclitaxel for injection (albumin bound), 150 mg of nedaplatin, and 200 mg of tislelizumab (September 18, 2022, October 15, 2022). On December 30, 2022, the chest and upper abdomen enhancement CT showed an increase in soft tissue of the left upper hilar accompanied by distal obstructive changes, which showed no obvious change compared with December 20, 2022. On December 30, 2022, a tracheoscopy showed that the upper lobe cavity was unobstructed and that nodular new organisms were visible at the opening. On January 6, 2023, the pathology report indicated a small amount of atypical proliferation of squamous epithelium in the left lung upper lobe opening neoplasm and the left main branch terminal tissue. On January 10, 2023, an upper lobe sleeve resection of the left lung of the left chest was performed. Postoperative pathology revealed pulmonary keratinized squamous cell carcinoma. There was no clear vascular embolism or clear invasion of the visceral pleura. On February 20, 2023, the patient’s creatinine was 98.9 umol·L^-1^. On March 8, 2023, one 200 mg dose of sintilimab was administered to the patient in the external hospital. In March 2023, the patient was self-consciously losing appetite and experiencing fatigue. On April 10, 2023, he was admitted to the outpatient clinic for “more than 2 months after the new auxiliary treatment of upper lobe cancer of the left lung, and 1 month with fatigue.”

**Figure 1:**
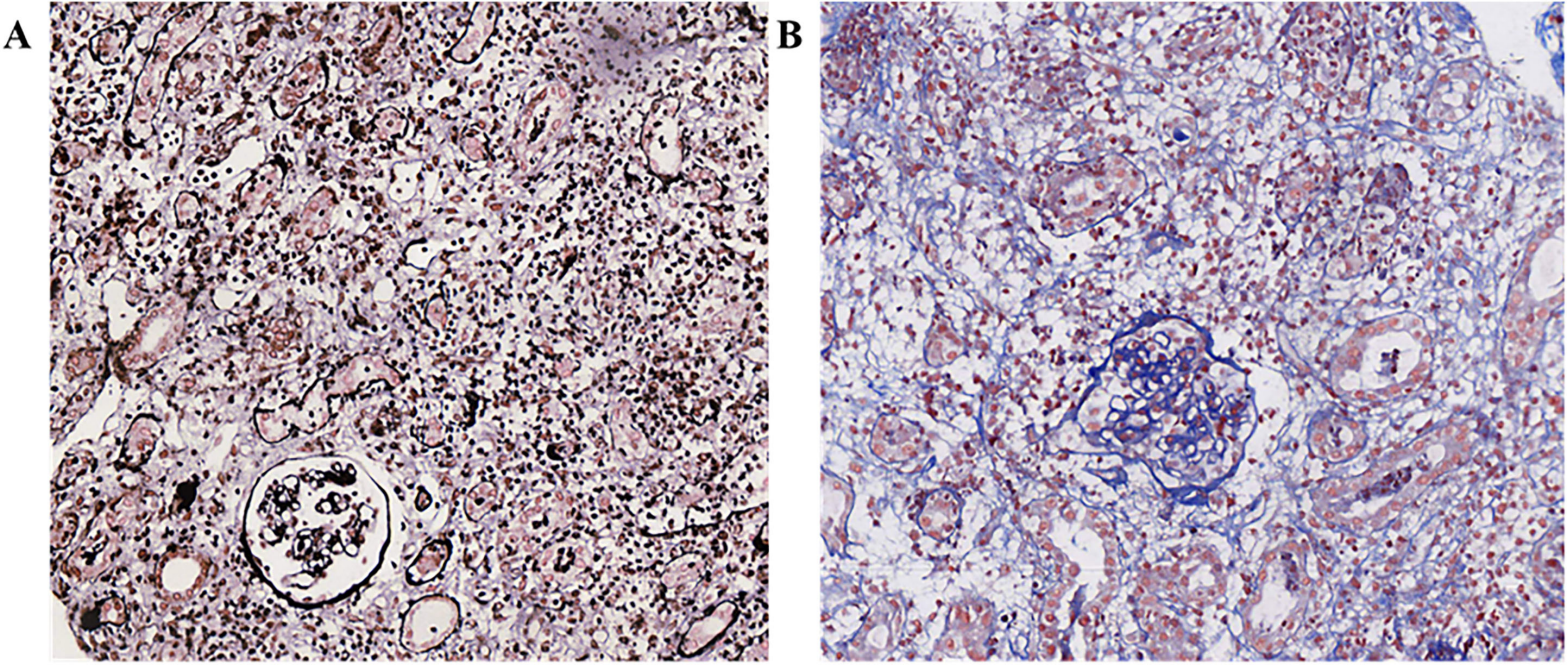
Renal pathological findings. **(A)** PASM+Masson X200. **(B)** Masson X200.

Diagnosis and treatment: On April 10, 2023, biochemical tests showed potassium 6.4mmol·L^-1^, creatinine 680.0 umol·L^-1^, urea 24.1mmol·L^-1^, uric acid 491.2umol·L^-1^. Due to the previous single cycle of sintilimab immunotherapy and clinical consideration of ICI-related kidney damage, the preliminary diagnosis was AKI stage 3 and hyperkalemia. According to the Common Terminology Criteria for Adverse Events (CTCAE) (1) version 5 and the Management of Immune-Related Adverse Events in Patients Treated with Immune Checkpoint Inhibitors: American The Clinical Practice Guidelines of the Society of Bed Oncology (2), the severity of kidney function abnormalities was graded 4. The urine volume on the same day was 410 mL, and continuous venous-venous hemodialysis filtration (CVVHDF) was carried out at 20:00 on April 10, 2023, and ended at 0:00 on April 13, 2023 (52 hours in total). The changes in the test indicators are shown in **Table 1**. On April 11, 2023, the patient’s appetite improved compared with a high potassium correction and type B sodium urine peptide precursor (NT-proBNP) 3570 ng·L^-1^. On April 12, 2023, the patient’s fatigue symptoms improved. On April 13, 2023, a kidney ultrasound showed 11.4×5.5 cm in the right kidney and 10.4×5.8 cm in the left kidney, and normal blood flow in both kidneys. Continuous venous-venous blood filtration treatment was carried out at 11:00 on April 14, 2023, and ended at 22:00 on April 15, 2023 (35 hours in total). On April 17, 2023, four premature renal injury tests showed urine microalbumin/creatinine 970.5 mg·g^-1^, α1 microglobulin/creatinine 403.7 mg·g^-1^, β2 microglobulin/creatinine 46.63 mg·g^-1^, and urine NAG/creatinine 72.4 U·g^-1^. There was no obvious edema on the patient’s body. The CVVHDF treatment was carried out at 11:00 on April 17, 2023, and ended at 12:00 on April 18, 2023 (25 hours in total). A kidney puncture biopsy was performed on April 18, 2023. On April 19, 2023, the patient had no lower back pain, no naked hematuria, and no nausea or vomiting. On April 20, 2023, the pathology of renal puncture (**Figure 1**) showed that the fluorescence was all negative, and 27 glomerulus (one glomerular sclerosis, two ischemic sclerosis, and the rest of the glomerular membrane cells and matrix mild proliferation) were observed under a light microscope. There was a loss of the brush border of the tubular cells. Renal interstitial edema, diffuse lymph cell, mononucleosis, and a small amount of eosinophil infiltration with fibrosis showed that interstitial invasive cells are CD3 (+), CD20 (scattered in +), and CD38 (scattered in +). The supplementary diagnosis was acute renal tubulointerstitial nephritis. Therefore, 40 mg IVGTT qd of methylprednisolone sodium succinate was administered (April 20, 2023–May 12, 2023). The CVVHDF treatment was began at 11:00 on April 20, 2023, and ended at 3:00 on April 23, 2023 (64 hours). On April 24, 2023, hemodialysis (HD) was filtrated for 4 hours. On April 26, 2023, the patient’s amount of urine increased, and dialysis was changed to HD three times a week for 4 hours each time (April 27–29, 2023). On May 2, 2023, the patient’s treatment was changed to HD twice a week for 4 hours each time (May 2–6, 2023). On May 8, 2023, before dialysis, the patient’s creatinine was stable at 350 umol·L^-1^, and the urine volume was acceptable. Dialysis was changed to once a week. HD was carried out for the last time once on May 9, 2023, for 3 hours and 20 minutes. On May 12, 2023, when the patient’s creatinine level was 299.3 umol·L^-1^, HD was stopped. The patient was prescribed prednisone acetate tablets (50 mg po qd) and discharged from the hospital.

**Table 1:** Changes in laboratory indicators.

Date	Blood potassium (mmol/L)	Serum creatinine (umol/L)	Urea nitrogen (mmol/L)	Glucose (mmol/L)	Uric acid (umol/L)	Urine volume (mL)	Weight (kg)
04-10-2023	6.4	680	24.1	7.9	491.2	410	52.5 (pre-dialysis)
04-11-2023	4.4	367.7	13.3	9.66	211.3	720	
04-13-2023	4.5	177.7	2.9	5.29	73.5	450	52.3 (post-dialysis)
04-14-2023	4.4	371.1	7	5.33	151.4	200	53.4 (pre-dialysis)
04-15-2023	4.1					160	52.9 (post-dialysis)
04-17-2023	4.2	399.6	7.6	5.51	175.1	150	
04-18-2023	4.2					100	53.4 (post-dialysis)
04-20-2023	4.7	465.9	10.1	4.73	242.8	150	55.3 (pre-dialysis)
04-23-2023	3.8	102.2	4.9	5.52	49.8	750	54.6 (post-dialysis)
04-24-2023							56.1 (pre-dialysis) 55.9 (post-dialysis)
04-26-2023	3.4	333.4	13.2	4.92	234	1100	
04-29-2023	3.2	348.2	16	4.62	302	950	52 (pre-dialysis) 51.7 (post-dialysis)
05-02-2023	3.2	354.9	17.7	4.87	233.3	1600	50.7 (pre-dialysis) 50.4 (post-dialysis)
05-05-2023	3.5					1250	
05-06-2023	3.9	359.6	14.7		222.4	1340	49.8 (pre-dialysis) 49.6 (post-dialysis)
05-10-2023	4.3						
05-12-2023	4.3	299.3	11.9	3.6	218.6	1850	

Out-of-hospital follow-up: Sintilimab was discontinued for tumor treatment, and no other treatment was administered. The chest CT scan on May 5, 2023, showed changes after the left lung surgery; there were multiple micro and small nodules in the right lung, and there was no obvious changes compared with the CT results on April 14, 2023. On May 19, 2023, at creatinine 273.8 umol·L^-1^, the patient’s prednisone acetate dosage was reduced to 45 mg/day. On June 3, 2023, the patient’s creatinine was 181.7 umol·L^-1^, and the prednisone acetate reduction was 35 mg. On June 30, 2023, the creatinine level was 161 umol·L^-1^, and the prednisone acetate reduction was 25 mg. On August 4, 2023, the creatinine was at 182.6 umol·L^-1^, and the prednisone acetate reduction was 20 mg. On August 18, 2023, the reduction of prednisone acetate was 17.5 mg. On September 8, 2023, the creatinine was at 196.5 umol·L^-1^, and the prednisone acetate reduction was 15 mg. On October 13, 2023, the creatinine was 195.1 umol·L^-1^, and the dose of prednisone acetate was reduced to 7.5 mg. On December 1, 2023, the creatinine was 238.7 umol·L^-1^, and prednisone acetate was discontinued on the same day.

### Ethics approval and consent to participate

The study was approved by the Clinical Research Ethics Committee of the Fourth Hospital of Hebei Medical University (2023-126-K92). The patient was informed about the study protocol and provided written informed consent to participate in the study.

## Discussion

### Case characteristics of AKI caused by ICIs

AKI is primarily based on the degree of creatinine elevation and changes in urine output. The internationally recognized grading criteria are currently based on the Kidney Disease Improving Global Outcomes. Previous studies have shown that the incidence of ICI-AKI ranges between 2.2% and 7.1% (9). Delayed onset, mild AKI, or AKI after the activation of ICIs are easily attributed to other causes, which may lead to an underestimation of the real incidence of ICI-AKI (10). A systematic evaluation study (11) showed that the combination of anti-PD-1/PD-L1 monoclonal antibodies with chemotherapy can significantly increase nephrotoxicity in patients with solid tumors. In recent years, studies have shown that the incidence of ICI-AKI is 1.4%–4.9%, and the incidence rate in some studies has reached 14%–18% (12). The most common pathological types of ICI-AKI reported in the study include acute tubular interstitial nephritis and glomerular disease, accounting for 93% and 7% of cases, respectively. Glomerular disease includes oligoimmune complex type GN, and vasculitis accounted for 27%; podocytopathies and focal segmental glomerulosclerosis accounted for 24%; and C3 glomerulopathy accounted for 11% (13). Clinical manifestations include kidney manifestations, such as proteinuria, hematuria, and sterile leukocyturia; other manifestations include an eosinophil count greater than 500 cells/mL. Multifactor logistic regression results show that hypertension, diuretic use, a baseline eGFR < 60 mL·min^-1^·(1.73 m^2^)^-1^, and extrarenal irAEs, are closely related to AKI occurrence in tumor patients treated with ICIs (14). The patient has hypertension, which is a high-risk factor for ICI-AKI. The routine urine analysis revealed a red blood cell count of 15.52 cells/μL, and the blood routine showed an eosinophil count of 0.02×10^9^/L, which is in line with the literature report.

On April 26, 2019, China issued the first “CSCO Immune Checkpoint Inhibitor-Related Toxicity Management Guidelines”; it was the fifth global guideline for the toxicity management of ICIs issued after publications by the European Society for Medical Oncology, the American Society for Immune Therapy of Cancer, the National Comprehensive Cancer Network, and the American Society of Clinical Oncology. All these guidelines recommend that CTCAE levels 3–4 AKI be discontinued and that ICIs be selected according to the condition. For level 3 adverse reactions, when the symptoms or laboratory indicators are restored to toxic reactions that are level 1 or lower, ICI treatment can be carefully resumed. For level 4 adverse reactions, ICIs should be permanently discontinued (15, 16). In addition, ICI-AKI treatment includes symptomatic treatment, glucocorticoids, immunosuppressants, and hemodialysis (17). According to the literature, immunosuppressive treatment of irAEs does not appear to affect the antitumor activity of ICIs (18). In our case patient had a grade 4 kidney adverse reaction. The clinical pharmacist recommends not using sintilimab at a later stage. Other similar ICSs should also be used with caution, but they need to be combined with the treatment of later tumors. According to the patient’s chest MRI scan, the tumor did not progress after the patient stopped immunotherapy.

### Literature review of cases and characteristics of AKI dialysis treatment caused by sintilimab

Common irAEs related to sintilimab use include fever, hypothyroidism, rash, pneumonia, fatigue, and decreased platelet count. AKI caused by sintilimab is uncommon. According to the literature, due to the lack of large sample studies, the incidence of AKI caused by sintilimab is unknown. AKI can occur as early as 3–4 h and as late as 12 months after the drug is administered (19). Researchers believe that irAEs usually occur within the first few weeks to months after the start of treatment but may also occur at any time, including after the cessation of immunotherapy (20).

Currently, only one case of dialysis treatment after AKI has been previously reported (8), and this paper reports the second case. The details of the patient reported in (8) and our patient are presented in **Table 2**. The patient in (8) underwent stage 3 AKI dialysis treatment for XX days and the patient in our case report underwent the same treatment for YY days after receiving sintilimab. The pathological types of the two patients were the same; both were treated with glucocorticoids and ceased ICIs treatment. The kidney function of the first patient (8) returned to normal. The renal function of our patient did not return to normal and developed into chronic kidney disease following the discontinuation of sintilimab. There was no tumor progression during the late follow-up of the two patients.

**Table 2:** Clinical characteristics of two patients with AKI requiring dialysis treatment due to sintilimab. .

Case	Gender	Age	Tumor diagnosis	Sintilimab regimen	Comorbidities	Time interval between AKI and the first application of sintilimab (d)	Peak creatinine (umol/L)	Renal pathology	Therapeutic measure	Outcome situation
Reference (8)	Male	56	Esophageal cancer	At 200 mg once every 3 weeks, medication once	Anemia	31	1,053	Acute tubular injury with small areas of interstitial nephritis	Dialysis twice, IV infusion of methylprednisolone at 60 mg/d; after 11 d, modified methylprednisolone was administered with an intravenous infusion of 40 mg/prednisone was changed 7 d	Renal function was normal and no further application of immune checkpoint inhibitors
									Oral administration of 30 mg/d, after 10 d, it was changed to prednisone orally for 20 mg/d	
This case	Male	71	Upper left lung carcinoma	At 200 mg once every 3 weeks, medication once	Hypertension, anemia	33	680	Acute renal tubulointerstitial nephritis	Dialysis 7 times, methylprednisolone IV infusion of 40 mg/d, after 22 d were changed to prednisone	Renal function did not normalize and no immune checkpoint inhibitors were applied
									Oral administration of 50 mg/d, after 7 d to prednisone for 45 mg/d, 15 days later, change to prednisone oral 35 mg/d	

**Table 3** summarizes findings from clinical trials of sintilimab, indicating substantial variability in the reported incidence of acute kidney injury (AKI) across studies. In clinical studies on, for example, classical Hodgkin lymphoma, gastric or esophageal junction cancer, lung cancer, recurrent or metastatic cervical cancer, liver cancer, and esophageal cancer (21–28) (see **Table 3**), the incidence of simple proteinuria is 4.8%–25% (21–23) and the incidence of proteinuria and creatinine is 2.2%–59.1% (24, 25, 27). The incidence of isolated creatinine elevation ranges from 1% to 3%, the majority of which are grade 1 or 2. Only six patients exhibited grade 3 or higher creatinine elevation (26, 28). The instructions indicate that among 2,461 patients who received 200 mg of sintilimab every 3 weeks, 568 were treated with sintilimab monotherapy and 1,893 received another treatment combined with sintilimab. In monotherapy cases, the incidence of proteinuria was 17.4%, and in combination therapy cases, the incidence of proteinuria was 22.2%. The incidence of AKI in 2,461 patients was less than 1%. The incidence of immune-related nephritis was 0.4% (11 cases); there was one case of grade 1 (< 0.1%), three cases of grade 2 (0.1%), seven cases of grade 3 (0.3%), and no cases of grade 4. Obviously, the incidence of adverse reactions of AKI above level ≥ 3 is very low.

**Table 3:** Clinical trials related to sintilimab.

Identifier	Tumor	Line	Drug	Phase	Number	ORR (%)	DCR (%)	PFS (m)	OS (m)	Number of kidney injuries	Percentage of people with kidney injury (%)
NCT02937116 (23)	Gastric/gastroesophageal junction adenocarcinoma	First line	IBI308 + XELOX	Ib	20	85	100	7.5	NR	2:00 AM	10:00 AM
NCT02937116 (24)	Non-squamous NSCLC	First line	IBI308&PC	Ib	21	68.4	84.3	12.6	18.9	1^a^	4.8^a^
	Squamous NSCLC	First line	IBI308&GC	Ib	20	64.7	100	6.5	15.4	5:00 AM	25^a^
NCT03629925 (ORIENT12) (25)	Squamous NSCLC	First line	IBI308 + GP	III	357	44.7 vs 35.4	NR	5.5 vs 4.9	NR	4vs1^b^ (0 vs 1^c^)	2.2 vs 0.6^b^ (0 vs 0.6^c^)
NCT03628521 (26)	Advanced NSCLC	First line	IBI308 + anlotinib	I	22	72.7	100	15	NR	13^b^(3^c^)	59.1^b^(4.5^c^)
NCT03794440	Advanced HCC	First line	IBI308+ IBI305	II	24	25	83.3	8.2	NR	10^b^	42^b^
(ORIENT-32) (27)			IBI308+IBI305 vs sorafenib	III	571	21 vs 4	72 vs 64	4.6 vs 2.8	NR vs 10.4	160 vs 34^b^ (20 vs 3^c^)	42 vs 19^b^ (5 vs 2^c^)
NCT03802240	Progressed NSCLC	First line	IBI308+ IBI305+PP	III	444	444 vs NR vs 25	83 vs NR vs 72	6.9 vs NR vs 4.3	NR	66 vs 35 vs 40^b^ (4 vs 0 vs 0^c^)	46 vs 24 vs 27^b^
(ORIENT-31) (28)			IBI308+PP+PBO2							4 vs 2 vs 3^d^ (2 vs 0 vs 0^c^)	(3 vs 0 vs 0^c^)
			PP+PBO1+PBO2								3 vs 1 vs 2^d^
											(2 vs 0 vs 0^c^)
NCT03150875	Squamous NSCLC	Second line	IBI308+ docetaxel	III	290	25.50 vs 2.20	65.50 vs 37.80	4.30 vs 2.79	11.79 vs 8.25	10 vs 4^b^	6.9 vs 3.1^b^
(ORIENT-3) (29)											
NCT03748134	Esophageal squamous cell	First line	IBI308+ chemotherapy	III	659	66 vs 45	90 vs 84	7.2 vs 5.7	16.7 vs 12.5	4 vs 2^d^ (4 vs 1^c^)	1 vs 2^d^ (1 vs 1^c^)
(ORIENT-15) (30)	carcinoma										

^a^Albuminuria; ^b^Both albuminuria and creatinine were elevated; ^c^Grade 3 or above adverse reactions; ^d^Increased creatinine; IBI308, Sintilimab; IBI305, Bevacizumab biosimilar drug; PP, Pemetrexed + Cisplatin; PC, Pemetrexed + Carboplatin; PBO, placebo; GC, Gemcitabine +Carboplatin; GP, Gemcitabine + Cisplatin; XELOX, Oxaliplatin + Capecitabine

### The mechanism by which ICIs lead to AKI

The mechanisms by which ICIs lead to renal interstitial damage include the following (20, 29):

1) When ICIs inhibit CTLA-4/PD-1/PD-L1, it releases the body’s “immune checkpoint.” While strongly activating the immunity of T cells to tumor cells, it also leads to the kidney’s tolerance of endogenous antigen decline, which triggers AKI. PD-L1 is expressed in renal tubular epithelial cell casts (RTECs), and ICIs combine with the checkpoint receptors expressed by the kidney to form hapten, which is called the “off-target effect.” These haptens are recognized by local dendritic cells and metabolized by renal tubular cells or bonded with carrier proteins after gaining immunogenicity. These bonds form forms an antigen–antibody complex and triggers an immune response (30, 31). Activated T cells recognize cross-reactive antigens in renal tissue and generate antibodies to mobilize typical antibody-induced hypersensitivity. In addition, activated T cells can infiltrate the renal parenchyma and release cytokines to produce inflammatory reactions.

1) Loss of tolerance induced by anti-PD-1/PD-L1 monoclonal antibodies leads to the reactivation of other drug-specific T cells. If the patient has used drugs that can cause acute interstitial nephritis (AIN), such as nonsteroidal anti-inflammatory drugs or proton pump inhibitors, or other drugs that can cause kidney damage, such as antibiotics (32, 33), after the above drug treatment, the activation of the immune response can produce drug-specific T cells. To some extent, with kidney damage, the aforementioned drugs can be used as exogenous antigens or semi-antigens as enhancers for the occurrence of ICI-related irAEs.
2) ICIs may be conducive to the production of autoantibodies against RTECs, membrane vascular cells, and foot cells, such as anti-dsDNA and antinuclear antigen antibodies (20, 34, 35). They have autoimmune reactions with specific renal autoantigens. Studies show that the treatment and interruption of ICIs and glucocorticosteroid treatment will affect the level of certain autoantibodies in the serum cycle, such as lupus-like glomerular disease and anti-dsDNA and antinuclear antigen antibodies, which are very similar to the phenotype of autoimmune lupus nephritis (34–36).

In our study, the patient’s sintilimab treatment led to a possible activation of AKI. It starts when sintilimab blocks PD-1 and leads to the destruction of autoimmune tolerance, triggering an autoimmune response to the kidney. In addition, the patient was taking aspirin, which can bind to renal tubules to form incomplete antigens. Applying ICIs on the basis of such drug exposure will further cause loss of T cell tolerance, prompt an immune response to the kidney, and increase the risk of AKI. Additionally, the patient’s age (71 years) and history of myocardial infarction were high-risk factors for AKI.

The immunohistochemistry literature (6) shows that CD3, CD4, and PD-L1 in the renal tubular stromata of patients are positive. It has been reported that patients with positive renal tubular PD-L1 expression are more likely to have adverse renal immune events in the process of receiving immunotherapy. Some scholars suggest that PD-L1 immunohistochemical may be an effective tool for distinguishing AIN related to anti-PD-1/PD-L1 monoclonal antibody treatment from other forms of AIN (37). Unfortunately, the renal pathology of this patient did not involve PD-L1 immunohistochemistry staining. In addition, with the application of new technical means such as proteomics, genomics, and drug poison metabolomics in the field of kidney disease, some new biological markers continue to emerge, which are important for the early AKI diagnosis and prognosis (38). These include glomerular functional markers (iron modulation), kidney injury or repair markers (calcprot Ein), IL-18, hepatocyte growth factor, neprilysin, renal tubular function or damage markers (kidney injury molecule-1), microRNA-21, and neutrophil gelatinase-associated lipocalin.

There are very few reports of post-dialysis treatment of AKI caused by sintilimab. Through the retrospective analysis of the case series in this article, we encourage healthcare providers to track the clinical manifestations of patients receiving ICI treatment in a timely manner, regularly monitor renal function electrolytes, and consider important risk factors for ICI-induced AKI, such as old age, underlying diseases, and combined medications. In future studies, PD-L1 immunohistochemistry may serve as a strong basis for determining the cause of AKI by ICIs. Through more further studies, the new biological markers are likely to serve as predictors of kidney injury, realizing early detection, close monitoring, and timely treatment of kidney injury to maximize the clinical benefits of immunotherapy for cancer patients.

## Data Availability

The original contributions presented in the study are included in the article, further inquiries can be directed to the corresponding authors.
